# Seniors’ campus continuums: local solutions for broad spectrum seniors care

**DOI:** 10.1186/s12877-020-01781-8

**Published:** 2021-01-20

**Authors:** Frances Morton-Chang, Shilpi Majumder, Whitney Berta

**Affiliations:** 1 CIHR Health System Impact Fellow (former), AdvantAge Ontario, Woodbridge, Canada; 2 Institute of Health Policy, Management and Evaluation, Dalla Lana School of Public Health, University of Toronto, Toronto, Canada; 3 Toronto, Canada

**Keywords:** Aging-in-place, Care continuums, Integrated seniors’ care, Seniors’ campus, Seniors’ village, Wrap-around seniors’ care, Community care, Long-term care, Retirement community, Seniors’ housing

## Abstract

**Background:**

As demand and desire to “age-in-place” grows within an aging population, and new areas of need emerge, governments nationally and internationally are focusing effort and attention on innovative and integrative approaches to health and well-being. Seniors’ Campus Continuums are models of care that seek to broaden access to an array of services and housing options to meet growing health and social needs of aging populations. The objective of this study is to increase understanding of this model and factors that influence their evolution, development, ongoing functioning and capacity to integrate care for older adults wishing to age in their own home and community.

**Methods:**

This research uses a comparative case study approach across six-bounded cases offering four geographically co-located components (mixed housing options, internal and external community supports, and a long-term care home) in various contexts across Ontario, Canada. Onsite in-person and phone interviews with senior campus staff (*N* = 30), and campus partners (*N* = 11), enhanced by direct observation at campuses explored historical and current efforts to offer health, housing and social care continuums for older adults.

**Results:**

Analysis highlighted eight key factors. Enabling factors include i. rich historical legacies of helping people in need; ii. organizational vision and readiness to capitalize on windows of opportunity; iii. leveraging organizational structure and capacity; iv. intentional physical and social design; v. broad services mix, amenities and innovative partnerships. Impeding factors include vi. policy hurdles and rigidities; vii. human resources shortages and inequities; and viii. funding limitations. A number of benefits afforded by campuses at different levels were also observed.

**Conclusion:**

Findings from this research highlight opportunities to optimize campus potential on many levels. At an individual level, campuses increase local access to a coordinated range of health and social care services, supports and housing options. At an organizational level, campuses offer enhanced collaboration opportunities across providers and partners to improve consistency and coordination of care, and improved access to shared resources, expertise and infrastructure. At a system level, campuses can address a diversity of health, social, financial, and housing needs to help seniors avoid premature or inappropriate use of higher intensity care settings.

## Background

### The changing face & pace of aging

As they age, most people desire to live as independently as possible and to receive care in their own homes and communities as their health needs, and those of the ones they care for, change and intensify [1–5]. In Canada, people aged 65 years and over now represent Canada’s fastest growing age group – a trend that is expected to continue for decades [6]. In the province of Ontario the number of those 65 and over is projected to almost double from 2.4 million, or 16.9% of population, in 2018 to 4.6 million, or 23.4%, by 2046 [7]. Healthcare and growing demands relating to increasing incidence of chronic and often complex conditions have been identified by Canadian seniors as important and ongoing concerns affecting their quality of life as they age. So too are concerns around adequate access to information, housing, income security, safety and security, social contacts and networks, transportation [8] in addition to informal caregiving supports and services [9, 10]. These concerns (demand factors) play an important role in determining an individual’s care destination; however, when, where and how a person receives care is also affected by other important “supply” factors including formal system capacity (e.g., state policies, funding priorities, service mix and volume, eligibility criteria, rurality, health human resources) and availability and willingness of informal systems to provide support (e.g., access to caregivers, care mix and volume, caregiver characteristics and resilience, social capital) [1, 11–14]. The dynamism amongst these demand and supply factors with the changing pace of population ageing pose challenges to providing coordinated approaches to equitable and accessible care and services to support “aging-in-place” as safely and independently as possible in communities of choice [1, 15–17].

### Canadian healthcare

In Canada, publicly funded healthcare is run by provinces or territories with financial support from the federal government to provide first dollar coverage for “medically necessary” hospital and physician care based on need, not means. To qualify, provinces and territories are bound by national terms and conditions outlined in the *Canada Health Act:* comprehensiveness, universality, accessibility, portability and public administration [18]. This has positively contributed to the medical care needs of Canadian residents, particularly high-needs seniors with complex conditions; however, there remains considerable variation between and within jurisdictions regarding if and how “extended health services”, such as homecare or residential long-term care, are funded and for whom. Social care, also characterized as community-based care, is another critical component to supporting and maintaining health and wellbeing of individuals with multiple needs. It is non-medical in nature and often reliant on social, environmental, and behavioural factors, as well as the availability of social support networks [19, 20]. Social care is not covered under the Canada Health Act and potential funding for such services also varies by jurisdiction.

In the province of Ontario, Canada, non-medical community-based support services span a range of programs and offerings including, but not limited to meals on wheels, transportation, friendly visiting, congregate dining, assisted living in seniors housing, adult day/enhanced dementia respite programs, and/or seniors’ wellness centres that provide educational, recreational, social and falls prevention programs. Publicly-funded homecare services are largely focused on medical health needs (e.g., post hospital stay, wound care, assistance with a bath) provided by contracted out professional (e.g., nursing, allied healthcare) and para-professional services (personal support healthcare workers) from community-based for-profit (FP) and not-for-profit (NFP) services provider organizations on a competitive basis [21]. Long-term care homes (LTCHs) are residential settings that provide long stay, respite and transitional convalescent care and services to adults in need of assistance with most or all of their daily activities including access to 24-h nursing and personal care beyond that which is typically be provided in a retirement home or assisted living services in seniors’ housing. LTCHs are subsidized by the province, but residents have to pay for accommodations [22].

The policy legacy of healthcare in Ontario has emphasized medical-focused care with less attention and resources focused on more preventative health and community-based coping care and supports [23, 24]. The siloed and fragmented nature of health and healthcare can pose great challenges for older persons with increasing health and social care needs, and those of informal caregivers to bridge the divides in care. Social factors such as poverty – with one in ten older Canadians living below the poverty line – increases the likelihood of experiencing medical and functional difficulties while making it less likely that people will be able to get the help they need when they need it [25]. Social isolation and loneliness, while not in itself a health condition, is now recognized internationally as a widespread and potentially debilitating condition among growing numbers of isolated older persons and linked to increased risks for increased blood pressure, heart disease, obesity, depression, anxiety, and cognitive decline [26, 27]. This in turn can increase the likelihood of default to higher cost hospital and institutional care even when more preventative home and community care options may be more appropriate and preferred [1, 19, 28–35].

In contrast, studies continue to show that seniors are better able to maintain independence and avoid inappropriate or premature use of hospital or LTCH beds when community-based homecare services and supports are: (a) available and accessible, (b), well integrated across and within the healthcare continuum (including social determinants of health addressed through community based social care services), (c) case managed, and (d) targeted to those at highest risk of nursing home care [1, 12, 30, 36–42].

The unprecedented economic and demographic pressures of an aging population, a corresponding rise in multiple chronic health and social needs, the decline of traditional social support networks, divergences across urban and rural access to services, and the persistence of costly system challenges means “business as usual” is not sustainable. This has governments across OECD countries looking to leverage existing infrastructure and care networks to develop new sector-spanning policies, roles and pathways to integrate health and social care and supports [1, 30, 43–45].

### Sustainable seniors’ care

A wide range of integrative programs and approaches have been identified in the literature that can be used to better “wrap” care around seniors wishing to receive care in their own communities including inter-professional care planning, coordination and system navigation, senior-friendly housing options with supports, community-based primary health care, and broader inter-sectoral approaches like age-friendly communities [3, 16, 46–48]. A model of care in Ontario that combines many of these approaches in one physical location is the Seniors’ Campus Care Continuum (also known as Seniors’ Villages). In this research, Campus Continuums refer to a single organization, or formal collaboration of a number of independent health and social services providers (e.g., community, housing, LTCH), that strive(s) to integrate the provision of a broad spectrum of seniors’ care (e.g., physical/psychosocial/social) within a defined geographic area. Campuses are often further characterized by offering different levels of care and a shared commitment/coordinated responsibility for wrap-around care (e.g., service planning, provision, case management, funding).

Seniors’ Campus Continuums (hereafter referred to as Campuses) align with the World Health Organization's (WHO) strategic objectives and with recent directions in Western Europe and in Scandinavia, where care for older people has been moving away from the development of stand-alone LTCH bed capacity towards more community-based or home care provision encouraging informal family support, implementing direct payments, and integrating housing, health and social care services [49–51]. Campuses that offer mixed-income housing affordable and rent-geared to income together with higher market rent and life lease housing aligns with the Canadian National Housing Strategy priorities to increase: affordable housing options with supports for those in greatest need, social housing sustainability, options for northern housing, sustainable housing for diverse communities, and a balanced supply of housing that leverages the blending of housing types to offset costs for social and affordable rental properties [52].

While campuses have existed in various forms over a number of decades in Ontario and elsewhere, there is a gap in the scientific literature regarding their inception and development into broader care continuums and enablers and challenges to maximizing their benefit and future development. This research aims to help fill this gap through an in-depth description and analysis of key factors that influence the evolution and ongoing functioning of seniors’ campus care continuums – in this case municipal and chairitable not-for profit campuses (NFP) – and their ability to generate a broad scope of health and social care for older adults.

## Methods

This study applied a qualitative comparative case study approach across six bounded cases (three municipal and three NFP seniors’ campuses in Ontario) to identify and understand key influencing factors on the evolution and ongoing functioning of expanded campus continuums in Ontario offering four key components – mixed housing options, LTCH beds, and community supports internal and/or external to the campus^1^ – and factors impacting on their ability to wrap care around their clients. Our sample stratification plan sought to maximize variation across several parameters known to influence organizational performance: organizational size (large or small), maturity (years since all four campus components offered), geography (urban, rural, northern), and cultural/ethnic/linguistic populations served. This exploratory comparative lens was used to develop an in-depth description and analysis of relevant dimensions to the evolution, design, and integration opportunities across a range of municipal and NFP seniors’ campuses through in-person or telephone interviews with senior campus staff (*N* = 30), and campus partners (*N* = 11) enhanced by direct observation. The cases were bound by time and place (2–3 days of on-site data collection including on-site interviews, campus tours, and researcher participation in campus activities) at six campus locations (*N* = 6) across Ontario [53]. Comparative cross-case analysis was selected because it allowed for the use of multiple information-rich cases to describe and understand how municipal and NFP campus continuums have evolved to their current configurations, the contexts they operate within, and implications for future advancement of the model.

### Literature review

Available research literature between 1975 to 2017 was reviewed, using OVID Medline and EMbase health sciences databases, to understand what scientific evidence was available on the campus continuum model that geographically collocates mixed-income housing with community supports (internal and external to the campus) and a LTCH and the ability of these components to work together to address a broad array of needs of seniors’ wishing to age in place. While several articles described various stand-alone components of campus continuums (e.g., benefits of care coordination, multilevel seniors housing, assisted living programs, inter-disciplinary care), none specifically described the evolution, development and ongoing functioning of campuses with the four geographically co-located components, nor how they attempt to work together to integrate care at different levels (e.g., individual, organizational, and the healthcare system). This gap in the research literature informed the direction of this study and areas of investigation. A semi-structured interview guide was developed to explore these factors as they relate to four-component municipal and NFP campus continuums – the focus of this research. (Please refer to Appendix A for overarching questions asked at each interview and additional suggested probes). A follow-up search was conducted in 2019 post data collection where new research is emerging on broader system configurations (e.g., municipalities) related to age-friendly-communities, but none specifically related to seniors’ campuses.

### Ethics approval and advisory group

Following research ethics approval for the project by the University of Toronto Research Ethics Board protocol # 35557, a Seniors’ Campus Advisory Group comprised of knowledgeable stakeholders including policy directors from AdvantAge Ontario, AdvantAge Ontario members with seniors’ campuses, academics, sector experts, and representation from the Ministry of Health and Long-Term Care (MOHLTC), and the Toronto Central Local Health Integration Network (TCLHIN), was created to provide advice and support in the advancement of Seniors’ Campus research including assistance with case study site selection, interview probes, reviewing preliminary findings, and knowledge dissemination. Advisory group members supported the selection of six case study sites of 16 submitting applications in accordance with specific selection criteria (diversity across key parameters for municipal and NFP campuses) which was explained to the group. An additional administrative review was preformed after case study selection by a selected case study prior to accepting to participate to ensure the study met with their own research ethics requirements.

### Case study sampling

A purposive sample was drawn from 33 potential case sites considered representative of municipal and NFP campuses in Ontario that offer the four geographically co-located components. Of the 33 campuses invited to express interest as a case study site, 16 confirmed their interest by submitting a template with a brief overview of their background, governance and each of the four components of their campus. This information was used by the advisory group and research team to inform case study site selection. Six representative case studies – three municipal and three charitable NFPs – were chosen to maximize variation across several parameters known to influence organizational performance including urban, rural, northern geography, variety in size, maturity, and unique ethnic/cultural/linguistic populations served (please see Table 1 for Campus Case Study Composition, Informants and Observation Period).

**Table 1:** Campus Case Study Composition, Respondents and Observation Period

Case Studies and Contexts at Time of Case Selection^**a**^ (***N*** = 6)	Interviews^**b**^ (***N*** = 34) Respondents (***N*** = 41)	On-site Interviews and Tours
**Northern (town)**	5 Interviews	2 days
Principal Provider: Municipal Campus Campus Size: 162 LTCH beds and 175 mixed housing units Housing: Social, Affordable, Market, Life Lease, LTCH Unique Populations: Francophone and Catholic Heritage Maturity: Four components offered > 15 years Visitor Hospitality Suite: No^c^	(4) Campus Staff (1) Campus Partner	
**Semi-Rural (town)**	8 Interviews	3 days
Principal Provider: Municipal Campus Campus Size: 143 LTCH beds and 139 mixed housing units Housing: Social, Affordable, Market, Retirement Home, Life Lease, LTCH Unique Population: Large Francophone sub-population Maturity: Four components offered in last 5 years Visitor Hospitality Suite: Yes^c^	(7) Campus Staff (4) Campus Partners	
**Urban (small city)**	5 Interviews	2 days
Principal Provider: Municipal Campus Campus Size: 128 LTCH beds and 198 mixed housing units Housing: Social, Affordable, Market, Life Lease, LTCH Unique Population: None specified Maturity: Four components offered > 15 years Visitor Hospitality Suite: No^c^	(4) Campus Staff (1) Campus Partner	
**Urban (large city)**	6 Interviews	2 days
Principal Provider: Charitable NFP Campus Campus Size: 198 LTCH beds and 227 mixed housing units Housing: Social, Affordable, Market, Cluster Care, LTCH Unique Population: Designated French language services provider Maturity: Four components offered between 5 and 10 years Visitor Hospitality Suite: Yes^c^	(5) Campus Staff (3) Campus Partners	
**Rural (town)**	5 Interviews	2 days
Principal Provider: Charitable NFP Campus Campus Size: 41 LTCH beds and 189 mixed housing units Housing: Social, Affordable, Market, Life Lease, LTCH Unique Population: German Mennonite Heritage Maturity: Four components offered > 15 years Hospitality Suite: Yes^c^	(5) Campus Staff (2) Campus Partners	
**Urban (city)**	5 Interviews	2 days
Principal Provider: Charitable NFP Campus Campus Size: 127 LTCH beds and 81 mixed housing units Housing: Social, Affordable, Market, LTCH Unique Population: Jewish Heritage Maturity: Four components offered > 15 years Visitor Hospitality Suite: Yes^c^	(5) Campus Staff (0) Campus Partner	

^a^ Each campus offered 4 geographically co-located components: Mixed Housing, Internal & External Community Supports/Programs, LTCH beds

^b^ Some interviews included more than one informant in the interview

^c^ The researcher stayed overnight in campus hospitality suites for those that had this amenity or stayed locally to the campus

### Case studies and interview respondents

While study sites and individuals interviewed are not identified to ensure confidentiality, due to the unique nature and configurations of Seniors’ Campuses in Ontario, campuses may be identifiable to knowledgeable observers in the sector. Senior leadership at each case study were alerted to this potential and were comfortable with possible recognition. Senior leadership of each case study helped to identify and recruit key campus staff and partner organizations interested and willing to be interviewed. Case study respondents (herein referred to as respondents) included a range of roles from each case study including but not limited to current or former chief executive officers, operating officers, finance officers, vice presidents, board members, and directors/managers of programs. Case study partners similarly brought a broad range of perspectives including, but not limited to, municipal housing, community agencies, universities, libraries, primary and allied care, local health integration networks, pharmacy services, and others. Please see Table 1 for further details on cases and respondents.

### Respondent anonymity

Respondents were provided with a consent form and letter of introduction explaining the research study and given the opportunity to ask questions prior to commencing the interview and contact information for any further questions or concerns. Written consent was provided by all interview respondents. Respondents in this study are not named and were each provided the opportunity to review their interview transcript prior to analysis. Subject matter was reported in aggregate where possible.

### Data collection methods, instruments and technologies

The primary investigator conducted 34 semi-structured interviews with 37 senior leadership and 11 campus partner organization respondents (some interviews had two or more respondents). The majority of the interviews were conducted in person and three respondents were interviewed by telephone for ease of participation. All interviews were audio-recorded and transcribed with respondents’ written consent and shared with the respondents for validation and or clarification (in some instances verbal consent was provided prior to a telephone interview and signed written consent form then provided to the principal investigator in follow-up to the interview). The principal investigator received a tour of each campus, ate meals with residents and participated in other resident activities (e.g., sing-alongs, meals, pub nights). Observations of campus activities were recorded in a field journal and incorporated to enhance interview probing questions and corroborate interview findings. Names of residents, family members, staff, or volunteers that were “observed” were not collected as agreed to in the ethics approval.

As outlined above, all respondents interviewed were given an opportunity to review their transcripts and make any necessary changes, clarifications or redact information. A slide-deck with preliminary findings was circulated to each interviewee for additional member checking to increase trustworthiness and credibility of the data analysis.

Senior leadership of the 10 campuses that had expressed interest in participating as a case study site, whose sites were not chosen, were invited to participate in a peer focus group (in-person or by teleconference) to review preliminary results and themes, and provide additional insights (ultimately, 7 of the 10 invited senior leaders attended). Initial findings were validated at this time and insights provided confirmed each of eight sub-themes as key factors experienced at other municipal and NFP campuses.

### Data sources and analysis

The primary data sources of this study were verbatim interview transcripts with campus senior leaders, management and key partners at the six case studies supported by supplementary sources (i.e., case selection summary templates,, interview notes, a journal log based on direct observations to inform probing questions, primary investigator participation in programs and activities with residents and additional documentation provided by case studies (e.g., brochures, activity calendars).

During early data collection and analysis, the primary investigator and one member of the research team (DB) reviewed four transcripts (two municipal and two NFP) and discussed emerging sub-themes based on the three previously identified overarching themes. The primary investigator met regularly with study co-authors to review emerging sub-themes and write up. The research team met with the advisory group at key internal checkpoints to share and discuss preliminary findings and areas for further analysis. Data analysis was guided by a hybrid approach of qualitative methods including a comparative case study approach using thematic analysis [54], which incorporates both an inductive approach that allows themes to emerge from the data [55] and a deductive a priori template-of-codes approach [56] based on observed gaps in the research on four component campus continuums. Audiotapes of the interviews were transcribed verbatim and data analysis was completed using line-by-line coding and constant comparative methods [57]. As transcripts became available following case study site visits, detailed within-case analysis occurred, followed by a thematic cross-case analysis with interpretations for the six cases combined. Generalizable lessons learned within the three broad cross-case themes were extracted and summarized [53] into **eight** sub-themes. As noted earlier, themes and related sub-themes were reviewed and verified in a peer focus group with other campus providers. A slide deck providing a summary of findings, similar to that which was reviewed by the peer consultation group, was sent to each respondent for review, to flag any concerns, or provide clarifications (none were received).

## Results

Factors that shaped the development and ongoing functioning of municipal and NFP seniors’ campus continuums and their ability to offer wrap around care for older adults (as described by campus senior leadership and organizational partners) were carefully considered across three overarching themes and organized into **eight** resulting sub-themes:

A. **Campus Inception and Development:**
  i. historical legacies
  ii. windows of opportunity
  iii. organizational structure and capacity
B. **Campus Design and Functions:**
  i. intentional physical and social design
  ii. campus service mix, amenities and partnerships
C. **Ability to Offer Wrap Around Care:**
  i. policy hurdles and rigidities
  ii. human resources shortages and inequalities
  iii. funding limitations

### Campus inception and development

In describing campus evolution, most case studies developed into a continuum incrementally over time with all six currently offering a broad spectrum of community-based health and social supports, mixed-income housing options and LTCH beds for seniors and older persons with ongoing care needs in one geographic location. Further enabling factors as described by case study respondents (campus providers and partners) are presented below. Please refer to Appendix B for supporting quotes to each theme.

#### Organizational legacies – addressing unmet and changing needs

Campus Respondents in this study described long organizational histories of caring for seniors and older adults with disabilities in need (e.g., local housing, healthcare). Respondents from the newest municipal campus and the most mature municipal case study noted legacies of helping vulnerable populations dating back to the late 1800’s starting as municipal “Houses of Refuge” (public and charitable organizations providing for social care, food, shelter and protection to the homeless or destitute) prior to becoming “Homes for the Aged” and then evolving into broader spectrum seniors’ campuses.

Respondents of the three NFP and the mature northern municipal campus described having deep ties to faith communities that recognized gaps in the system for local seniors, particularly those with specific cultural, religious and/or linguistic backgrounds. The mature NFP campuses began as community driven enterprises where faith leaders sought to address housing and care needs within the contexts of their respective Mennonite and Jewish religious and cultural heritage. The Mennonite campus began its journey in the 1970’s when a collective of Mennonite churches developed independent seniors’ housing to address local housing needs for seniors in their community. The Jewish campus began as a Home for the Aged in a setting that respected Jewish culture and Kashrut (dietary laws). The northern mature municipal campus, and the newest NFP designated Francophone campus, while not faith-based, benefited from strong foundational support and ongoing relationships with the Catholic Church (e.g., advocacy, access to resources) and the Francophone communities in which they operated. The northern municipal campus began as a district Home for the Aged to meet the needs of Francophone seniors in their district and foundational staffing was provided by Catholic nuns and a priest who resided and presided over their onsite parish. The newest NFP campus, an official designated French Language provider, began its evolution as a hospital run by the Sisters of Charity in the late 1800’s that has since evolved to include LTCHs, community-based care and most recently seniors’ housing.

Respondents from mature campuses described the 1970’s to the 1990’s as a time when many seniors in residential LTC often had lighter care needs than that which was offered in those settings currently (e.g., many were still driving yet in need of light monitoring, care, or simply safe affordable shelter). Campus respondents attributed expansion into more fulsome continuums as a means to address widely varying levels of need for seniors and adults with disabilities wishing to age in their communities (local and/or faith-based). Since that time, campus and partner respondents alike highlighted that acuity and complexity of care for residents in their LTCHs had increased over time as well as in their supportive housing (SH) and Assisted Living Services (ALS) programs.

Respondents from the municipal campuses in this study noted that where provincial law sets certain requirements on municipalities to offer LTCH options (separately or jointly) in southern Ontario, the expansion into continuums was not a legal requirement. However, municipal respondents noted that co-locating a range of mixed housing and community support options including LTCH options in one physical location was considered both a responsibility to their community members (taxpayers) aligned with municipal “seniors’ strategies” but also met broader goals and commitments to developing “Age-Friendly Communities.”

Respondents from the NFP campuses similarly noted that expansion into seniors housing and additional community services was a natural extension of their mandates to serve their local and/or identified community (heritage/religious/linguistic). The newest NFP campus continuum, originally a hospital and LTCH provider, highlighted the direct impact a lack of housing and care options was having on their hospital bed use and estimated approximately a third or more of patients in one of their hospitals did not belong in hospital, but rather in a LTCH or supported in an assisted living services (ALS) program in the community. These alternatives would not only improve seniors’ well-being, but create system efficiencies. (See Appendix B). Case study respondents across all six case studies emphasized the bedrock of campus inception to expansion was visionary leadership (past and current founders, administration, board members, local councils and faith communities), with high political acumen (e.g., sitting at the ‘right’ tables; working collaboratively community partners) and willingness to take risks (financial, organizational).

#### Organizational vision and readiness for windows of opportunity

Respondents noted expansion to broad care continuums required capitalizing on opportunistic policy windows and government incentives (e.g., development and redevelopment funding) and being “shovel ready” when they arose. Respondents from mature campuses highlighted stimulus funding for housing through a combination of federal, provincial and municipal grants and ongoing operating subsidies in the 1980’s and early 1990’s, greatly supported their expansion of affordable housing for independent seniors with light care needs and the development of “elderly persons” wellness centres, and SH programs. They further noted that expansion efforts became constrained by the mid-1990’s with a change in provincial government and new policy directions towards developing LTCH bed capacity. (See Appendix B).

Study respondents from newer campuses highlighted that in the last decade new federal and provincial capital funding opportunities and incentives across different levels of government and ministries helped to seed interest and ability to expand affordable housing offerings. (See Appendix B).

Respondents from all of the campuses described how they leveraged existing infrastructur**e** (e.g., LTCH) as an “anchor” from which to develop other housing options, supports and services. Some indicated a goal of up to four or five lighter or more independent housing types per LTCH bed with ranges of services available would best address growing needs. Expansion into a full continuum was most often described as incremental and reliant on opportunistic funding incentives by government. In contrast, the newest municipal campus was able to develop each of the four components of campus continuums being investigated in this study across one period of time by capitalizing on a redevelopment opportunity for their aging standalone LTCH. Respondents from each of the newer campuses described benefitting greatly from discussions on lessons learned and site visits with more mature campuses across the province to observe and gauge fit for their own context and roll-out.

Campus respondents noted the importance of offering a blend of mixed-income housing options to address issues around access and availability for varied financial abilities to pay. Options included social housing (subsidized rents for low income individuals), affordable housing (generally 80% of market value rent), market rent housing, and for most case studies, life lease agreements (residents own their own units but must sell back to the organization when moving or in the event of death).^2^ (See Table 1**)** All campus respondents identified access to affordable housing options as critical. Some campus respondents highlighted that many low income seniors were just above the threshold to qualify for a limited supply of social housing on campuses but could not afford to pay even “fair” market rent. One mature NFP campus demonstrated great commitment to social housing in keeping rent geared to income options for one of their legacy buildings past the required timeline by the municipality to do so.

The newest municipal campus developed a retirement home option for those with means as an additional offering to address higher care needs along the continuum (e.g., on site nursing, three meals per day) than are available by government funded ALS programs that are limited to a relatively small percentage of seniors’ housing residents (~ 20%) in many of the case studies. Campus respondents described while retirement home options are only possible for seniors with financial means, that this was worth considering as a potential option to further expand continuum offerings while also providing a campus with a wider economic base to support fixed overhead costs, cost recovery for the subsidized units (well below market prices) and enhance stability of the campus and its programs.

#### Organizational structure and capacity to expand

Campus respondents described having similar corporate structures and governance arrangements with key entities of the campus (e.g., LTCH, housing, foundation) generally having their own respective boards or advisory committees with oversight through an overarching corporate board and cross pollination across the different boards and committees/councils. Campus corporate services (e.g., administration, human resources, finance) were largely centrally administered and viewed as a means to improve operating efficiency, sharing knowledge and skills across the organization and for standardizing global practices and policies affecting quality of care, service and cost.

Respondents noted campus development activities “*were not for the faint of heart*” and described them as highly resource intensive to undertake (e.g., capital, time, finances) from idea to implementation. Leveraging existing campus infrastructure (e.g., number of licensed LTCH beds), organizational capacity (e.g., internal expertise for feasibility studies, land procurements, partnership development, renderings, project management) and adequate cash reserves to put upfront against campus (re)development were described as key enabling factors for expansion opportunities. (See Appendix B).

Municipal campuses and larger NFP organizations were described by respondents as more able to draw upon in-house knowledge, expertise and capital to manage campus planning, development and ongoing functioning as needed (e.g., human resources departments for advice, procurement and property offices to help with capital development plans). This minimized the need to contract out for required skills and project management. Securing funding posed challenges, even for large campuses, as funders could have difficulty understanding the interconnectedness of the multiple components within a coherent continuum of care and when projects needed to be “value-engineered” to work within set limits and timelines.

### Campus design and functions

Campuses were described by campus and partner respondents as being “*more than a place to live”* and having “*a real village atmosphere”*. They highlighted the importance and intentionality of interconnected campus components as a means to offer diverse opportunities for social and civic engagement by campus residents and local community. Campuses further expanded access to health and social networks through intra and inter-organizational coordination of services, offering important onsite amenities and development of creative partnerships to improve integration of services both vertically and horizontally. Key enabling factors to campus design and function are described below:

#### Intentional physical and social design

Campus architectural built environment was attributed as a key factor to the “vitality” and ongoing functioning of campus life and activities. Each campus noted intentional strategies to ensure physical interconnectivity (e.g., covered above ground linkages, connecting corridors, cleared outdoor walkways) and social interconnectivity (e.g., shared programing, shared amenities, common rooms) across campus components. Such connectivity was considered critical to the health and social well-being of seniors to facilitate planned and spontaneous opportunities for physical exercise (alone, with staff, friends and/or family), and ability to socialize with others in different areas of the campus. Safe linkages provide greater ease of passage for patrons, particularly those using mobility devices or with vision impairment. They also reduce seasonal risks for heat exhaustion with high temperatures in the summer, or simply not having to put coats and boots on deep cold in the winter. Where physical linkages were not as convenient as others (e.g., going to separate buildings through underground corridors) campuses worked hard to make these spaces appealing (e.g., local artwork), senior friendly (e.g., seating between areas) and purposeful (e.g., spaced utilized for hair salons, cafés) to promote their use. (See Appendix B) Physical connectivity was also seen to provide and enhance opportunities across the campus for participation in spontaneous and planned activities, and address the potential for loneliness and social isolation. Respondents of a mature municipal campus described careful consideration given to the redesign a large common room in their LTCH auditorium to make it more inviting to other residents of the broader campus (e.g., sky lights, wide screen television, a small pub, an ice cream parlor, ample seating) and a central area to attend collective programming and social events. Similarly, a respondent from another mature municipal campus described recently converting a former greenhouse in their LTCH into a popular lounge area where, given the higher proportion of housing residents than LTCH residents, instituted a policy that housing residents need accompany a LTCH resident to enjoy the area with them. This has provided family and friends of LTCH residents a greatly desirable space to enjoy visiting in.

Case studies located in smaller towns were largely populated with people from the local community and tended to have multiple residents with shared histories (e.g., attended the same schools, religious institutions or service clubs). It was not unusual for residents in the (semi) rural or northern campus to be related in some way (e.g., siblings, cousins, in-laws, grandparent) to other residents or with a campus staff (past or present). Respondents noted benefits in having family and friends in close proximity including an increased ability to maintain kinship and support for one another. Proximity, freedom of movement across campus buildings, and familiarity to campus staff and programs were all noted to promote greater uptake of onsite respite opportunities (ADPs, respite LTCH bed) by informal caregivers and greater opportunity for visits if onsite placement of a spouse/sibling/friend to the campus LTCH occurred.

Respondents noted that while all campuses aimed to be inclusive in their programing and offerings to potentially all residents, tensions could sometimes develop when offering housing that crosses socio-economic spectrums and different abilities (e.g., physical, cognitive, developmental). They further noted that different housing options that provide enhanced finishes or selling features (balconies, appliances) could contribute to potential divides; some felt these were necessary to attract seniors willing to pay extra for these features and offset costs for the lower cost rentals, while others felt offering the same to all avoided unnecessary distinctions of disparities. Proactive strategies described by campuses to address these issues included the development of tenant advisory boards to work together in developing communal activities (e.g., potlucks hosted in different common rooms across the campus) and enhancing opportunities for leisure, social engagement or volunteering regardless of income or abilities (e.g., communal libraries, garden spaces, party rooms, billiard rooms), shared amenities (e.g., worship space), and community wide activities open to campus residents and local community (e.g., live entertainment, special events, religious services).

All campuses noted socio-economic distinctions tended to fade as residents of the campus participated in collective activities and got to know and look out for one another. Respondents further noted that collective activities and shared spaces were also important mechanisms reduce the potential for loneliness and social isolation not only for campus residents, but also for clients attending on-site day programs, recovering patients in convalescent care in campus LTCH beds, and visiting family and friends of LTCH residents wishing for opportunities to engage in or simply seek a change of scenery. Commonly described campus offerings (recreation, events, amenities) are listed in Table 2.

**Table 2:** Common Shared Recreation, Amenities, Events and Volunteer Opportunities

Recreation Opportunities^**a**^	On-Site Amenities^**a**^	Events and/or Volunteer Opportunities^**a**^
• Bingo • Pub nights • Art classes • Choir • Line dancing • Religious services • Off-site outings • Woodworking • Shuffle board • Wellness centres – gym, therapy pools	• Health related clinics/labs • Hair Salon • General store/Tuck Shop • Community gardens • Library • Restaurant/Bistro/Café • Laundry • Common spaces for planned and spontaneous activities • Hospitality Suites^b^	• BBQs • Live entertainment • Bazars • Golf Tournaments • Tuck Shop • Friendly Visiting • Family Councils • Board Committees

^a^Offered at many campuses

^b^ On campus hospitality suites are available in many campuses for a modest fee to accommodate out of town family and friends to increase access and affordability in the promotion of resident visitors. Campuses without hospitality suites noted informal arrangements could potentially be considered/were offered with local hotels to provide discounts to guests specifically visiting residents of the campus

#### Service mix, amenities and partnerships

Campuses were described as having the ability to coordinate access to a continuum of care in one geographic location for a blend of low to high needs and often underserved populations. In contrast to service-by-service delivery approaches where clients have to navigate multiple services and providers on their own, campuses were designed to consolidate resources to offer “one stop shopping” where clients receive help to access the most appropriate services and supports from a continuum inclusive of community supports, housing options, and LTCH beds The blend of independent living and housing with supports was viewed as crucial to maintaining individuals with a range of lower to higher needs as independently in the community for as long as possible. Specifically, legacy supportive housing (SH) programs in Ontario (pre 2011), assisted living services (ALS) programs (2011 onward), cluster care (CC) and retirement home (RH) living were all noted as beneficial options to access progressive levels of support for those in need of assistance but not requiring LTCH level care. Respondents identified issues with current long-term care waitlist practices for clients needing to transition out of housing to onsite LTCH beds – to be discussed later – which had campus staff stretching their SH, ALS and RH programs to try and bridge time to secure an onsite LTCH bed when their application became available and try to avoid the need for a client to move off campus to another LTCH.

Campuses offered a similar core set of programs and services (see Table 3 for a summary) assigned by a care coordinator or case manager. Examples provided included lighter coping supports for independent living (active seniors who do not require care support but may get peace of mind from 24 h security, and the option to purchase light housekeeping, programs and meals), to addressing higher levels of need through government funded case managed services and ALS program supports (personal care, medication and meal monitoring). Some programs were staffed by the campus and others by community partners renting space such as Adult Day Programs (ADPs). The northern municipal campus was unique in that they did have and ADP offering for seniors, but it was supported off-site in another community in need of seniors’ services. Many campus respondents expressed concern that by comparison to other sub-sectors, the community support services (CSS) sector was poorly funded. When delivering CSS services or partnering to do so, this disparity could impede abilities to serve more people in need (wait lists) or raise fees as program expenses increased. Many campuses included basic service packages as part of their rental agreements (e.g., minimum purchase of congregate dining meals per month, telephone, cable) with an option to purchase additional services. Service packages were described as helpful mechanisms to monitor and address safety (e.g., daily security checks), well-being (e.g., gauging gait, balance or cognition during programs), social isolation (e.g., opportunities to dine with others) and nutritional needs of seniors (e.g., healthy meals with no meal preparation required), while also helping to off-set costs in provision of providing these programs onsite.

**Table 3:** Common Campus Home and Community Care Programs

*I = Available Internally to Campus Residents = I; E = Available Externally to Local Community*						
Campus	Meals on Wheels	Day Program^**a**^	Active Living Centres/Wellness Programs^**b**^	Falls Prevention Programs/ Physio-therapy^**c**^	Congregate Dining	Supportive Housing/ Assisted Living^**d**^
Northern Mature (Municipal)	I & E	Not onsite		I & E	I & E	I
Semi-Rural (Municipal)		I & E	I & E	I & E	I & E	I
Small Urban Mature (Municipal)	I & E	I & E	I & E	I & E	I & E	I & E
Urban (NFP)	I & E	I & E		I & E	I & E	I & E
Rural Mature (NFP)				I & E	I	I
Urban Mature (NFP)	E	I & E	I & E	I & E	I & E	I

^a^ Programs that provide structured and supervised activities for frail and socially isolated seniors and individuals with cognitive impairment offered during the day, evening or overnight

^b^ A centre with programs and services that promote socialization, recreation, physical activity, learning, friendships, community involvement and independent living

^c^ Group exercises and falls prevention education to help seniors stay healthy and active

^d^ Programs for a set number of clients in housing units (differed across campuses) deemed eligible for intensive case management and care coordination of personal care and other supports based on Standardized Resident Assessment Instruments (RAI-CHA)

Co-location of the various campus components and consolidation of resources were described as allowing for greater efficiencies and maximizing economies of scale which benefited clients and provider organizations (e.g., bulk purchasing for the entire campus; maximizing use of existing infrastructure to the broader community). For example, case study sites often purchased and sold utilities back to campus residents at a significant discount to what they would otherwise pay individually. For residents, this removes the need for them to have to organize directly with external companies and avoid strangers in to set up the utilities. For utility companies, respondents noted this helped to avoid any confusion in navigating installation across the campus and having one payer. In another example, study respondents noted economies of scale in shared offerings of the LTCH kitchen with other food programs (e.g., Meals on Wheels, congregate dining) which extended food security and improved nutrition within and beyond campus walls to the broader community (See Appendix B).

Campus staff noted some contracting out for certain tasks (e.g., pharmacy services, the use of agency staff to cover personal support worker (PSW) or nursing shifts) was more practical than others (e.g., housekeeping and maintenance).

Campus respondents described the impact of their municipal and NFP contexts on the way they are able to operate and manage money. Municipal campus respondents described levies as an important factor affecting their ability to build reserves into their operations. Municipal respondents noted levies were used to maintain service (e.g., automatic door openers, elevators, damages from wear and tear) when housing contracts were complete, to address any shortfall in revenue and to provide wages and benefits that attract and retain qualified staff. However, should campuses be able to accumulate a surplus “for a rainy day”, municipal campus respondents noted that it is not always viewed as managing well, but as having levied too much and requiring continuous negotiation with the municipality. (See Appendix B).

Respondents from NFP campuses also reported having to exercise caution around the amount and manner in which they would plan, fundraise or carry a surplus in order to maintain their charitable status (e.g., not making profit and reinvesting revenue into campus operations and care). (See Appendix B).

Municipal campus respondents in smaller communities noted that municipalities work collaboratively with the local community and the private sector, paying careful attention to respect the balance of the private sector. Respondents from NFP campuses also reported working closely with private and public sector organizations to encourage the development of local echo-systems for the benefit of residents of their campuses and local community. (See Appendix B).

Campus and partner respondents highlighted the need to be knowledgeable across a vast array of policies and legislation they operate within, some of which were common across all components, and others more specific and targeted to care setting. In offering a wide array of health and social care, campuses also worked with different ministries (e.g., Ministry of Health and Long-Term Care, Ministry of Municipal Affairs and Housing, Ministry of Community and Social Services, Ministry of Seniors Affairs and Accessibility). Campus respondents of the newest NFP campus noted an additional level of accountability to an overarching hospital board and having to adhere to hospital-based policies. Please see Table 4 for an overview of key legislation and policies in which campus components operate in Ontario.

**Table 4:** Key Policies and Legislation Ontario Campus Continuums Operate Within

Campus Feature	Provincial Legislation or Policy
Independent Seniors Housing	• Residential Tenancies Act 2006. • Housing Services Act, 2011
Assisted Living/Supportive Housing	• Home Care and Community Services Act, 1994 • Assisted Living Services for High Risk Seniors Policy, 2011
Adult Day Programs	• Patients First Act, 2016
Wellness Centres	• Seniors Active Living Centres Act, 2017
Retirement Homes	• Retirement Homes Act, 2010 • Residential Tenancies Act, 2006
Long-term Care Homes	• Ontario Long-term Care Homes Act, 2007
Hospital	• A Public Hospitals Act, 1990
Foundation	• Canada Revenue Agency Guidelines • Not-for-Profit Corporations Act, 2010 • Individual Gift Agreements with Philanthropists
Unions	• Labour Relations Act, 1995 • Collective Agreements
Common to All	• Building Code Act, 1992 • Employment Standards Act, 2000 • Fire Protection and Prevention Act, 1997 • Health Protection and Promotion Act, 1990 • Human Rights Code, 1990 • Municipal Regulations and By-Laws • Personal Health Information Protection Act, 2004 • Quality of Care Information Protection Act, 2016 • Workplace Safety and Insurance Act, 1997

Source: https://www.ontario.ca/laws

Volunteerism by campus residents, local community, family members, and school placements was noted as playing a pivotal role in the ongoing functioning and vibrancy of all campuses. Volunteer opportunities ranged from direct resident contact (e.g., friendly visiting, delivering meals on wheels on site, helping at ADPs, portering/accompanying people to and from social activities) to event planning and fundraising committees (e.g., auto shows, barbeques, gardening, organizing bazars) and/or formal committee membership (e.g., tenant councils, family councils in LTCHs). Respondents noted volunteering provided seniors, particularly those living in campus housing, an important sense of purpose and natural opportunities to socialize and reduce loneliness and isolation; proximity increased their ability to help. Campus respondents also highlighted the many volunteer hours staff themselves provide in supporting extra-curricular events like dances or barbeques and other activities.

Innovative campus partnerships and arrangements with community organizations, government bodies, academic institutions, local providers (medical and non-medical) and local businesses enhanced campus offerings and helped them to serve as “community hubs” for which the broader community is encouraged to share in programs, supports, amenities and events. Co-location with partner organizations often mitigated the need for travel for campus residents and local seniors affording savings in transportation costs, and built important on-site linkages to clinical (e.g., primary care, audiology, blood labs) and non-clinical services (e.g., banking hours/machine, libraries; general store, pharmacy services, social programing, restaurants). An important practical limitation for campuses was as their scope grew – so too did issues with a lack of parking for clients, staff, visitors, and volunteers. Please see Table 5 for examples of key partnerships with providers and local businesses (some with lease arrangements) at many of the campus study sites.

**Table 5:** Key Partnerships and Supportive Arrangements

Government Partners	Community Partners	Clinical Intervention Partners ^*^	Academic Partners**
• Municipal (housing, paramedics) • Regional (Local Health Integration Network homecare) • Provincial (Ministry of Health and Long-term Care, Public Health, Ministry of Housing and Municipal Affairs, Infrastructure Ontario) • Federal (Canadian Mortgage and Housing Corporation)	• Community Care Agencies • Hospitals • Community Health Centres • Primary care • Alzheimer Society • Community Living (serves people with developmental delays) • Mental Health Agencies • Pharmacies • Faith Communities • Local Businesses • Community Programs (choirs) • Shelters	• Audiology • Chiropody • Denture care • Primary care • Phlebotomy lab • Physiotherapy • Pharmacy Services	• Colleges • Universities • School Boards

* Visiting clinics, contracted providers, adjacent health facilities or lease agreements

** Internships (e.g., PSW, RPN, RN, Recreation, Culinary), research opportunities and volunteer opportunities (e.g., high school/co-op)

### Ability to offer wrap-around care

Respondents at each campus described three common factors that impact upon their ability to offer wrap around care across a full continuum: policy hurdles and rigidities, human resources shortages, inadequate funding.

#### Policy hurdles and rigidities

Respondents noted that while all campuses conceptually have the ability to offer a full continuum of care to support people’s needs as they change or intensify – sometimes temporarily, sometimes permanently – each campus operated under and across multiple policies, legislation and sectoral standards that could pose barriers to do so seamlessly. An issue that emerged across all case studies was the management of LTCH waitlist policies, which in Ontario are under the control of the local regional government and applied using an equity lens for all in need of a bed across the province. Priority status is not given to residents living in campus housing for a LTCH bed on their campus. While legislative provisions are made for spousal reunification in LTCHs if one person is already in a LTCH bed (in the context of this study – a LTCH bed on campus), respondents noted that no such provisions are made for onsite priority of other family members living on campus (adult children with physical, cognitive or developmental disabilities) who would benefit from being in close proximity. Respondents highlighted that in cases where campuses provide for specific cultural/religious/ linguistic needs, clients in campus housing with these backgrounds (e.g., Francophone speaking; Jewish heritage) “in principle” had a greater chance of getting into the onsite LTCH, but would often wait long periods of time on the list for an opening.

Campus respondents expressed that while they understood the value and importance of equity-based policies for LTCH placement, they pointed to the lack of priority status as “incongruent” with the purpose and perceived benefits of care continuums to maintain a person in their familiar community of choice. Respondents noted the continuum was the draw for many seniors to move to a campus despite being informed at time of application and entry there would be no guarantee that an onsite LTCH bed would be available if and when spaces opened at time of need. Mature campus respondents further noted this practice was counter to campus founders’ original intentions to support people across transitions at the most frail and vulnerable point in a resident’s life and for those that had been in campus housing prior to the change in wait list policies, moving across all components of the campus was an expectation. (See Appendix B).

Campus respondents described resulting consequences of transitions from campus seniors’ housing to an external LTCH setting as including increased burden for frail partners/family/ friends to attempt to monitor and maintain connection (e.g., transportation costs and energy for traveling distances), and severed relationships with familiar staff and settings.

Campus partners acknowledged and sympathized with these concerns, yet also described experiences of their community-dwelling seniors and caregivers in great need of a LTCH bed. They described off-site seniors as not having the same level of monitoring, support and access to services and amenities available to those in campus housing waiting for a bed making their needs equally and potentially more pressing to prevent additional stress or burnout by caregivers. Campus and partner respondents both noted that the campus interconnectivity and amenities supported uptake to their programs (e.g., Adult Day Programs, Wellness Programs, Congregate Dining, Volunteering) and enhanced transition experiences to LTCH placement with greater familiarity of setting, and ability to maintain social relationships (visitors across the components). (See Appendix B).

For individuals arriving to the campus LTCH from hospital or community as a “crisis placement” (a term that indicates an inability for people to manage independently at home and/or those with a distressed caregiver), campus respondents noted that, after having been rehabilitated, some of the placements had the potential for lower intensity care elsewhere on campus. They further noted that few opportunities were available to shift such residents into campus housing programs for a “trial” without losing their LTCH bed, and no assurances to be next on the LTCH waitlist if the shift was unsuccessful. Study respondents noted frustration that some people can end up receiving care in the “wrong” places and additional observations that some “crisis placements” appeared to be in less need of higher level LTCH care than some seniors receiving support and care on their ALS programs.

While campus respondents had little control over funding restrictions or LTCH placement waitlists, they were able to manage their internal wait lists for housing – with some restrictions on rent-geared-to-income – to help residents move across other campus settings (e.g., life-lease to apartments with supportive housing or cluster care) based on need and already being on campus – not chronology. Internal wait lists were also described as helpful when considering entry of a life partner or disabled adult child to campus housing from the community when their spouse/parent moved to the campus LTCH home from the community. (See Appendix B).

#### Human resources shortages

Human resources shortages experienced by campuses ranged from PSWs and nurses to culinary and allied staff. These shortages were noted as a more prominent concern in the north and rural regions, but in all cases by comparison to stand-alone community programs and LTCHs, campus challenges were less acute. All campus respondents indicated that provision of in-house PSW care was preferred by housing residents to provide greater reliability and staff familiarity. The impact of PSW shortages on external community support service agencies to the campus, was noted as impacting on their reliability and consistency of staff than in-house PSWs. Francophone campuses expressed that shortages are further impacted in seeking to find bilingual staff. At the time of data collection, some campuses were exploring becoming sole contracted providers for the provision of all government funded PSW services for campus residents. (See Appendix B).

Campuses described current strategies to ensure coverage for required services and accountability agreements including: i. the use of external agency staff to cover shifts which for some could become expensive (e.g., equivalent to paying staff at overtime rates); ii. strategic contracting with another community support agency to provide care on a semi-regular basis as a means to better plan for and cover vacation and sick time for staff in different areas of the campus, avoid paying overtime, and prevent staff burnout; iii. Hiring developmental services workers (DSW) instead of PSWs for one ALS program to provide an additional level of training for the increasing acuity of community residents with health and social care needs; iv educational placements and internships with students from local colleges and universities (health care, maintenance, culinary) to host, train and potentially hire new staff. (See Appendix B).

Campus respondents described relatively low staff turnover in administrative positions but greater fluctuation for front-line LTCH staff. Despite higher pay rates in LTCHs for these positions than in the community, some campus respondents described a trend of staff transferring into housing positions citing the attractiveness of lower ratios of patient to staff. Campus respondents also described a historical trend of staff moving to hospitals where PSWs are paid higer wages (similar wage rates as the LTCH sector pays registered practical nurses (RPNs), making it tough for campuses to compete for PSWs). Respondents from the northern municipal campus noted that recruitment was particularly difficult in the north and remote communities as compared to their urban counterparts.

#### Funding limitations

At the time of data collection, respondents noted that budgets for community support services (CSS) in Ontario had not increased for approximately a decade (prior to the 2018–19 budget). In contrast, respondents noted increases in hospital and LTCH funding over the same period. Campus and partner respondents described frustration that CSS were “*treated as less essential to other healthcare offerings despite their value and ability to offer high level care at the same or lower cost than if they were to be placed in institutional long-term care.”* [CR1]. (See Appendix B).

Community-based ALS programs were described as able to provide enhanced support to people in lower care settings (their own apartments) – often delaying or avoiding the need for placement to LTCH. Respondents noted that ALS programs experienced policy rigidities that limited the intensity level of clients they were formally able to serve when in practice respondents noted that campus ALS programs often supported people with a range of moderate to very high needs; particularly when clients were deemed appropriate for LTCH placement. They did so through a range of services and supports on campus and partnering with the regional authority for nursing and other professional care services to address client preference to live and die in ones’ own home when adequate support was available to do so. Historical funding configurations impacted the nimbleness of ALS programs to respond to need and forced the creation of waitlists for some case study sites. (See Appendix B).

Campus respondents also described experiencing stress when unable to bring senior residents experiencing need onto their ALS programs due to funding constraints and resulting waitlists. In cases where clients’ needs exceeded the support ALS could offer, campus respondents described working with the regional government to partner in care or suggesting to residents and informal caregivers to arrange private pay options (campus staff or outside agency) to “top-up” services, or to help bridge gaps while waiting for a LTCH bed. (See Appendix B).

Respondents noted that social and affordable housing offered on campuses were essential to age in the community, but expensive to offset, particularly given the growing number of seniors in need of subsidized housing. Campuses noted market rent, life lease, and retirement home living options could help to do so and to extend care continuum offerings, noting that careful attention would be paid to reinvest any surplus back into the campus – a key factor to meeting NFP and charitable status. The newer municipal retirement home offered additional options that included the ability to offer short term stays (one to three months) for seniors in need of seasonal help (e.g., winter stay to avoid risk of falls/snow shoveling), or a trial stay for permanent consideration. These options were noted as addressing organizational needs (suite capacity) and system needs (assisting hospital discharge planning with transitional alternatives to convalescing or LTCH placement).

Campuses utilized additional opportunities to secure funds for programs and services including fundraising events; however, for those without formal foundations, fundraising could be disruptive to other administrative activities. As discussed earlier, small revenues could also be generated through the inclusion of minimum service packages as part of rental agreements separate with direct reinvestment of funds in campus programs.

## Discussion

Findings of this study highlight a constellation of facilitating and impeding factors that influence campus development and their ability to bridge divides in health and social care for seniors and older adults with varied levels and types of need (e.g., physical, social, financial, housing). The key implications of our findings for policy decision makers and provider organizations, and considerations for future inquiry are discussed below.

### Campus inception and development

#### Building on historical legacies

Each campus case study identified using existing infrastructure (e.g., LTCH, existing housing) as “anchors” on which to build broader continuums. They further highlighted the critical importance of having forward-thinking responsive leadership deeply committed to the health and welfare of local seniors in addressing unmet health, housing and social care needs of their local community**.** Campus founders, senior leadership and governance**,** were described as “being on the pulse” of both community need but also monitoring the policy environment to anticipate and navigate “windows of opportunity” (policy directives, funding incentives, community interest and support) ready to leverage existing assets (funding, facilities, human resources, and partnerships) to capitalize on the opportunities to meet current and emerging needs. Key policy changes in housing and LTCH (re)development in the 1980s-, the early 1990s, and again over the past decade have afforded opportunities to campus developers to set the pace and direction for further development, including expansion to broad mixes of housing types and ratios (social, affordable, “fair” market, retirement, life lease), inclusive design (physical and social connectivity) and expanded service-mix based on local context (e.g., community needs, supply and buy-in). New opportunities are emerging as Ontario undertakes the restructuring of its health care system. Key initiatives include building or redeveloping 30,000 residential LTCH beds [59] and creating a province-wide network of Ontario Health Teams (OHTs) accountable for “delivering a full and coordinated continuum of care to a defined geographic population” [60]. While LTCH (re)development funding is directed at LTCH beds, such funding may incent broader thinking by standalone LTCHs and Seniors Housing into campus development and further expansion by existing campuses. Organizations may also benefit from leveraging compatible incentives through municipal, provincial and national housing strategies to address calls for more affordable housing for vulnerable seniors and other underserved populations [52].

#### Co-location for person-centred care

By co-locating a broad range of services, programs and providers to clients, campus continuums can tailor more integrated care plans and service packages that provide progressive options for changing client and carer needs. Co-location and partnering enhances opportunities for communication, collaboration, case management, shared infrastructure and expertise across the continuum to achieve “person-centred care” inclusive of progressive health supports (e.g., medication monitoring, bathing) and non-medical coping care (e.g., opportunities for social connectedness, wellness activities and amenities). Studies have shown that access to such coordination across health and social sub-sectors improves the ability of individuals and their informal caregivers to remain independent and avoid inappropriate or premature use of higher intensity care settings [1, 12, 30, 36–41, 61]. Accessible and available caregiver programs and supports like those offered at campuses are also identified by a growing body of literature as preventative in nature and reduce the likelihood of developing illnesses from burnout (good for people), and mitigate inappropriate utilization of acute care resources (good for the larger system) [62, 63].

Many benefits were noted to individuals, campuses and the system through the development of partnerships with key stakeholders (e.g., primary care providers, denturists, pharmacies, banks, hair salons) that increase access and proximity to important health and social services for residents and local seniors while also reducing the need to travel long distances to access them. Campus connections with educational institutions were also symbiotic relationships with all parties benefiting from job placement, research, and volunteering opportunities. These creative arrangements generate new resources and opportunities for campuses and their broader communities.

Building a critical mass of services and resources in a single location and centralization of services can create operational efficiencies and generate economies of scale that can be reinvested into campus programs and services; in turn this can extend campus services further outward to contribute to the health of communities beyond their borders (e.g., off-campus assisted living, meals on wheels, and on-campus day programs, wellness centres, and educational programs and services offered to seniors in public libraries). Combined, these facilitating mechanisms align with critical success factors for integrated systems of care (intra/inter-organizational, inter-professional, inter-governmental, and inter-sectoral collaborations and coordination) outlined in the literature which again support person-centredness, organizational efficiencies, and avoidance of people unnecessarily ending up in the “wrong places” in the system [3, 16, 30, 37, 46–48, 64].

### Campus design and functions

#### Campuses as vibrant “age-friendly” communities

Co-location and collaboration were further enhanced by intentional physical connectivity of the architectural built environment and shared programing. Covered walkways and linkages across buildings were critical to mobility and freedom of movement for seniors of different abilities to exercise, participate in programs, socialize, check on relatives and friends, and to facilitate volunteer opportunities. Shared public spaces and amenities further encourage social engagement and mutual support in planned and spontaneous activities (recreational or social) by campus residents, external community members, and visiting family and friends. Campus design and service mix were also observed to provide many benefits to individuals and the system by connecting people early or “upstream” to preventative services and coping supports which can help to prevent or reduce the negative impact of social isolation and loneliness on mental and physical health as identified in recent literature [65, 66].

As noted earlier, campus continuums strongly align with the WHO’s expanding *Age Friendly Cities* movement [67] to enhance seniors’ local participation and provide measures to give them opportunities to live safe, independent and dignified lives [1, 43, 44]. They further align with the *Global Strategy and Action Plan for Aging and Health* [16, 68] (now driving “seniors strategies” in Ontario) – a movement committed to action on healthy aging, developing sustainable and equitable systems and providing age-friendly integrated care over the longer-term in affordable and accessible settings (home – LTCHs) that meet the needs, rights and preferences of older populations [8–10] [16, 68].

While case study respondents highlighted many advantages to providing a broad mix of services and housing, they noted that were they to construct a campus de novo, they would be more mindful of tensions that can arise due to differences in physical design of units or financial capabilities across mixed income buildings. To preempt or mitigate these tensions and address feelings of “other” sometimes experienced by lower income residents, new campuses may consider proactive strategies to minimize the occurrences or need for these differences, provide basic service packages for all residents to engage in joint activities (e.g., congregate dining, excursions), and ensure representation from the various housing components on tenant committees.

### Ability to offer wrap around care

While respondents identify many advantages of campuses regarding their ability to coordinate and offer integrated care they noted that campuses still face significant challenges related to policy hurdles and rigidities, human resource shortages, and funding inequalities across sub-sectors.

#### Policy hurdles and rigidities

While campuses generally operate under one provider, by virtue of working across multiple sectors (health, housing, social care) they confront an array of sometimes conflicting laws, regulations, funding arrangements and accountability requirements. Indeed, conceptually campuses can offer access to a full continuum of care from independent living to LTCH placement, campuses in Ontario operate in a fragmented health care system consisting of numerous institutional “silos” (e.g., housing, community supports, LTCH, hospital) each with their own eligibility criteria, service limits and accountability requirements. This can obstruct smooth transitions between care settings even on campus. One of the greatest challenges described in the findings by campuses respondents was around LTCH wait lists. External control by regional health authorities applied through an equity lens, or based on “crisis” placements, did not provide priority status to campus residents. Those who have lived on campus for long periods may still have to move elsewhere when they require LTCH placement resulting in difficult transitions and a loss of sustaining friendships and social networks. Future options for consideration may include more hybrid solutions that accommodate percentages or extenuating circumstances beyond spousal reunification.

It is worth noting that while LTCH waitlists can pose barriers for many in need of such high level care, many campus housing residents are able to actualize aging and dying in place without LTCH placement (e.g., dying of natural causes; palliative care in their home). Limitations to scope of practice for SH/ALS staff, program eligibility criteria, and ALS wait lists were seen to impede optimization of these campus resources to allow all campus residents to receive care. Expansion of scope and eligibility may help to alleviate Alternate Level of Care (ALC) pressures in hospitals (occurrences where people no longer need acute care in hospital but are unable to return home or move to a LTCH). This is an important consideration – particularly as the province of Ontario seeks to address solutions for “Hallway Healthcare” in hospitals and the desire of seniors to age-in-place [69]. Policy makers should consider leveraging enabling age-friendly frameworks aimed at increasing the ability of campuses to “flow” people smoothly to the most appropriate place on the care continuum rather than allowing people to default “upward” to high intensity care options (hospitals and LTCH beds) because of a lack of “lower level” prevention and maintenance services.

#### Human resources shortages and inequities

Like other providers, campuses face persistent staff shortages which have campuses working hard on recruitment activities and retention strategies. Consolidation of human resources is a strategy with the potential to facilitate staff working seamlessly across different program areas of the campus. In practice, however, many factors could interfere with consolidation including the earlier-mentioned legislative requirements by sector/program or differing rules of collective agreements (e.g., differences in job categories, responsibilities, compensation levels). This can result in one staff member having different contracts with the same employer, at different pay rates and reduce campus flexibility to reassign staff where needed as emergent situations arise. In trying to share workers across programs and services, campuses still face difficult equity issues since workers in different areas may experience historical pay disparities (e.g., PSWs in LTCHs typically earn better pay and benefits than those working in community settings). Creating policy to address historical wage inequities and funding parody across sectors would seem to have strong potential to address both recognition for the value of the work and make the community more competitive with other sectors.

A strategy used by all case studies was partnering with educational institutions to inspire and train future workers while noting the time it takes for a person to complete training, pay for it, juggle a part-time job and/or childcare to do so were additional barriers to even developing a workforce from which to recruit. A future consideration for policy makers and providers is to institute paid apprenticeships for skilling up a robust PSW workforce and addressing issues of wage parody for similar work in different settings.

#### Historical funding limitations

By virtue of working across sub-sectors, campuses are affected by the legacies of managed competition for home and community care services. In the mid-1990’s the Ontario government placed downward pressures on wages and working conditions for PSWs in the sector while making hospitals and LTCHs more attractive locations for higher paid work [23]. As noted above, this policy legacy continues to impact current day PSW and RN shortages and campuses’ ability to meet staffing requirements [70]. Despite this legacy, campuses described struggling less than independent CSS agencies in having a centralized hub with consolidated services and human resources. A further implication of sub-sectoral divides experienced by campuses at an organizational level were discrepancies in government funding where their LTCHs (and in one case study their hospitals) would receive modest annual increases yet their community programs and support services received little to none. Frustration was expressed by study respondents that, despite CSS potential to offer preventative and maintenance to high-level care at the same or lower cost to the system (and individuals) than if people landed prematurely to a LTCH bed or an avoidable  stay in hospital (ALC, Emergency), funding and policy directives for CSS were not as highly valued nor considered as essential as other healthcare settings. While policy-makers emphasize the importance of supporting people “closer to home,” disproportionate funding would seem an important consideration for future investments in delivery of care for seniors over the longer term; conceivably it would be more expensive to build a LTCH bed than support care in one's own home.

Campus continuums would also seem to benefit from more flexible, “person-centred” funding mechanisms. In countries like Germany, Japan, and the US, integrated client funding envelopes, based on assessed needs, are used for the care of older persons. For instance, PACE (Program of All Inclusive Care for the Elderly) models in the US, the international “gold standard” for the care of ageing populations, receive capitated funding for high needs clients based on what it would cost to place them in a nursing home [1]. Since PACE is responsible for all care, including the costs of hospital and LTCH admissions, the incentive is to keep people well and independent. Ontario is now experimenting with “bundled funding” where health providers are paid a single fee for a specified package of care to an individual [71]. Bundled funding could potentially be extended to cover all services for campus clients to incent innovative “before-the-fact” care that avoids unnecessary hospitalization and LTCH placements. Funding strategies to actualize seniors’ desires to age-in-place may include enhancements to current program funding (e.g., top-up services, expansion for those on ASL program waitlists), investing in more cluster care options (small scale congregate living designed specifically to meet the monitoring and vulnerability support needs for people with cognitive and or physical declines, and optimizing a hub and spoke model to fan out of additional supports to more clients in the neighbouring community to help mitigate or avoid premature LTCH placement where possible [72].

### Strengths and limitations

A strength of this study is its contributions to gaining a better understanding of campus diversity and functioning through the comparison of municipal and NFP campuses offering a range of mixed housing options, community supports internal and external to the campus and LTCH bed yet varying in several parameters (e.g., size, geography, special populations). The dynamic interplay and integration opportunities between campus providers and co-locating them in one geographic location with similarly dedicated partners is something that has received little attention, although of great relevance in the current context of ageing populations.

While this research captures many lessons learned about the history and ongoing management of Seniors’ Campus Care Continuums from multiple perspectives (representation from senior management of programs and services, environmental services and valued partners such as Local Health Integration Networks, municipal housing, primary care), key informants interviews did not seek to interview matching/equivalent roles at each case study – although this did occur in some instances – or with for-profit campuses.

An important lens that is missing and would be beneficial to include is direct resident perspectives. While the researcher was permitted to observe resident activity during site visits and enjoyed meals with residents which reinforced and confirmed many themes developed in this paper, the voice of people living on campuses and supported by campuses in the community is not captured. An ethics review that would include respondents drawn from potentially vulnerable populations would have been prohibitive by extending the length of time to commence and complete the study within the timeline of the fellowship.

## Conclusions

To achieve the promise of seniors’ campus care continuums as fully integrated, affordable, accessible and quality care settings it is important to understand how they take root, the opportunities and challenges that they face in bridging the health and social care divide, and prospects to maximize their benefit across varied contexts. While various components of campuses have been studied on their own merit, this study fills a gap in the scientific literature on how campuses attempt to integrate these components – often in a system that is not facilitative of their efforts to do so – to maximize their benefit for variety of health, housing and social care needs at many levels.

At an **individual level** municipal and NFP campus continuums have the ability to address diverse physical, cognitive, social, respite, spiritual and financial needs of individuals with progressive declines and their informal caregivers through co-location of mixed housing options and supports. Their intentional design provides interconnectivity for greater ease of movement to services, programs and amenities and reduces need to travel distances for services, while enhancing opportunities for social participation and creating a sense of community. Co-location and familiar staff can further contribute to better uptake of respite programs by caregivers – an essential backbone to the healthcare system – to help prevent or delay stress and burnout.

At an **organizational level**, campus continuums serve as valuable employers in the local economy and a “hub” of supports, services and entertainment for the local community. Campuses create efficiencies in service delivery through centralizing administration and maximizing economies of scale. The intra- and inter-organizational workings of a campus can contribute to fewer and smoother transitions and serve as rich environments for educational practicums and volunteer work.

At a **system level**, campuses continuums offer enhanced system coordination and case management of health and social care options for older adults wishing to age-in-place in their own communities and help to avoid unnecessary or premature utilization of more intensive system resources.

Despite many advantages to these integrative care continuums, they continue to experience policy hurdles, human resource shortages and historical inequities in CSS funding. Moving forward, while population aging may occur primarily as the result of demographic trends outside the reach of policy-makers, considerable degrees of freedom exist in deciding how to use scarce resources in response to this growing population and their desire to age-in-place. New windows of opportunity for scaling and spreading the campus model may open based on external pressures in the form of National and Provincial Seniors’ Strategies, Dementia Strategies, Affordable Housing Strategies and Age-Friendly Community Strategies as evidenced in Canada and other countries, and growing international agreement that such strategies are crucial. Findings from this study are timely as Ontario aims to restructure its healthcare system, including the building and redeveloping of 30,000 residential LTCH beds and creating a province-wide network of Ontario Health Teams (OHTs) accountable for “delivering a full and coordinated continuum of care to a defined geographic population.” The results of this study and potential solutions to barriers, supported by international literature on age-friendly innovations, point to Seniors’ Campus Continuums of Care as uniquely positioned to respond to rising needs and advance transformative system change.

### Future research

This research contributes to a broader understanding of the evolution and development of seniors’ campus continuums and lays a foundation for future exploration using different lenses (e.g., public health lens, social ecology) and capturing other important perspectives (e.g., resident lived experience). Future research may look to explore measures of resident health, well-being, quality of life, and level of satisfaction with the model including the impact of having to move off-site for a LTCH bed if that situation arises. Future policy making may also benefit from scholarly comparisons of client outcomes and/or cost effectiveness with for-profit models and/or with stand-alone LTCHs and seniors housing.

## Data Availability

The raw data are not available to members outside the research team and will not be shared beyond the study team as per our ethics approval.

## Appendix A

### Interview Protocol

1. **Review Letter of Introduction and Consent Forms and provide any necessary clarification on the study details, requirements and consent process.**
2. **Proceed to Interview Questions**

I. **Background: Please describe why / how your campus evolved to its current array of programming, services and supports.**
  Suggested Probes for exploration (dependent on respondent’s role):

- What is the history of your campus? Was it planned/strategic or would you describe it as more serendipitous?
- Did you look at other campus models?
- How did you initially establish /undertake activities for a campus of care?
- When did linkages between/amongst services begin to be recognized as important (and why considered important at the time)?
- Formal or informal partnerships? If yes, how does this occur?

II. **Corporate Organization and Overall Governance: Please describe your campuses current corporate organization and overall governance structure. (*****Indicate we will focus on the individual components of the campus later in the interview.*****)**
  Suggested Probes for exploration (dependent on respondent’s role):

- Corporate structure
- Please describe the campus’ relationship with the broader local community
- Does the campus offer any community outreach within and/or beyond your campus borders (e.g., personal support, transportation)?
- How do you monitor the well-being of campus residents across the continuum of your campus? In the community?

III. **Programs and Services across the Campus Continuum: Please describe the current array of programming, services and supports offered through your Senior’s Campus, either directly or through partnerships, and the nature of required arrangements to offer them (e.g., key development and implementation processes).**
  Suggested Probes for exploration (dependent on respondent’s role):

- Describe the service X (housing, supportive housing and assisted living, LTCH, community services, amenities, etc.) within the campus and, if appropriate, the local community.
- What are the relevant policies and legislation within which programs operate?
- Briefly describe how the programs and services are funded and how they relate to the overall entity (required funding arrangements, sources, and accountability arrangements to offer the continuum of care)?
- How are services managed across the continuum with the campus and local community?
- Could integration of services be improved?

IV. **Moving Forward (all respondents): How might one better facilitate the ability of campuses to maximize their benefit for their residents/tenants and local community?**
  **Suggested Probes for exploration (dependent on respondent’s role):**

- Lessons learned from campus development and implementation experience?
- Necessary partnerships, outcomes and potential scale or spread?

V. **Thank respondent(s) for participating in this important research.**
VI. **Remind respondent(s) they will receive transcripts for review shortly.**
VII. **Provide contact information for Principal Investigator Dr. Morton-Chang if there are any questions that arise over the course of the research.**
  Frances Morton-Chang, MHSc, PhD
  CIHR Health Systems Impact Fellow
  AdvantAge Ontario and The University of Toronto
  Email: frances.morton@utoronto.ca

## Appendix B

### Supporting Quotes for Identified Themes

A. **Campus Evolution**
i. **Organizational Legacies – Addressing Unmet and Changing Needs**

*“About one to two-thirds of our [clientele] were seniors in our hospital and LTCHs … So we thought ‘that’s our niche’. There’s a dire need [for affordable housing and assisted living] and here’s an opportunity that really could continue the legacy of the sisters, which was to address the most vulnerable in the community [and] to build something that we can transfer [people out of hospital]. It’s good for seniors’ quality of life but it’s also good for the system as a whole because if you look at what it costs per day to sit in a hospital bed versus being in assisted living [in the community] … You’re comparing $600 a day to $50 to $70 a day.”* [CR1]

*“The Ministry [conducted a] pilot project … of 102 hostel beds and a 40 unit apartment building … What they cared about was ‘how do we best serve the seniors in this community given their health levels?’ … So when the Ministry came out with new housing opportunities in 1992 … the board was ready. They had the building designed, the land prepared...It was all on paper.”* [CR2]

ii. **Policy Changes afforded Windows of Opportunity**

*“Funding was from the Ministry of Housing [in] the early ‘80s – and was really, in today’s world, kind of a gift. It financed 50 year mortgages and there were provisions in for rent supplement for people that needed it … So it gave you a nice mix of incomes in terms of modest income through to folks that were pretty well off. And there was pretty quick pick-up. People could understand the notion of ‘I don’t need a nursing home but living at home doesn’t work for me anymore’.”* [CR3]

“…*we received a grant for the affordable housing units as part of the stimulus… This was in the time of the sub-prime crash. The federal government matched provincial dollars but it was administered through the city. The city didn’t contribute to that, [but] they… gave us breaks on some of the developmental charges [like]… permit fees.”* [CR1]

iii. **Organizational Structure and Capacity to Expand**

*“There are a lot of requirements. And I’m talking binders, different levels [of bureaucracy], we had to submit to [the municipality], and resolutions … and my director of Facilities was an engineer and that made things easier…between him and I, we project managed the construction. We didn’t have to hire out…and the financing … was a challenge [which] required a tremendous amount of paperwork… [and] borrowing millions of dollars. And we had the infrastructure to do it. We had the staff…a CFO, a corporate controller. And I was just trying to think, you know, if a smaller organization was trying to do this, it would have been difficult.”* [CR1]

B. **Campus Design and Functions**
i. **Intentional Physical and Social Design**

*“*…*deliberate creation of a common gathering space between two formerly separate buildings which encouraged people to sit down, to share in community with each other… structures with a design that can bring people together naturally.”* [CR5]

ii. **Campus Service Mix, Amenities, Partnerships and Arrangements**

*“Our kitchen makes the meals for the LTCH, for supportive housing, for the Meals on Wheels program, for the [partner organization] adult day program, and then caters meals for various different events and groups that use our community hall... And having the additional meals helps us to have a more substantial staff group in our kitchen for the purposes of providing the meals for all the other events and activities that we get involved with.”* [CR6]

*“But to get anywhere near close to what the building condition assessment says we need [for future repairs], we need to put more money into our reserves over the next 10, 20, 30 years to be able to replace the whole roof, to be able to replace all the windows, to be able to do all those things we’re going to need to do in 25 years. And, being in the municipal sector, we would need to go to council and explain ‘we need to up our reserves by say 2% a year’ or figure out how we can do that internally in this building.”* [CR7]

*“We’re getting into the foray now of designing a new LTCH…and a community hub…[which] might mean, maybe, there’s some commercial space in that community hub that generates revenue that supports what’s going on here [but] we have to be careful what we do on campus that we don’t jeopardize our not-for-profit and charitable status.”* [CR5]

*“So we all work together…I think it’s a social responsibility that you have with your private sector…We [as a municipal campus] offer social housing and have to balance as to how many units we should have so not to negatively affect the private sector.”* [CR2]

C. **Ability to Offer Wrap Around Care**
i. **Policy Rigidities and Enablers**

*“There is no question that access to a program like [SH/ALS] can prevent or postpone placement into long term care. We see it happen all the time.”* [CR4]

*“*… *if they are on my assisted living program and suddenly they’re on a LTCH wait list… we do our best to hang onto the resident until that [onsite] bed is available or until they end up in crisis and they have to go somewhere else.”* [CR8]

*“If they have to go to the hospital from our apartments, then convalescent care, and go back to their apartment, the campus allows that flexibility for people to move right or left on the continuum, to a point. And that point is the long term care placement.”* [CR5]

*“Yes, you’d be moving, and that’s a stress at any point in your life. But at least you’re still here [on campus] with people you know.”* [CR9]

*“Some of our day program clients also access the music programs and the respite programs upstairs in the long-term care home...We have quite a few families [living in the community and campus housing] that use long-term care respite [care beds] because then they can get a week or two weeks break at a time. …So, it’s a bonus to our clients because they get used to that atmosphere. So if they are moving into the long-term care home, families tell me they find it’s a little better transition [for these clients].”* [CR10]

*“We have an internal wait list such that if people who already live here in the condos and want to live in another place within the village, they can ‘jump’ the wait list. For example we have people that are on the wait list in the condos to come to a one-bedroom unit in this building. So there is continuity in that as their care needs change, they don’t have to really change their address and their community.”* [CR11]

ii. **Human Resources Shortages and Innovation**

*“[current staff are] tired of working short as well. So we need to balance… that there are not enough PSWs coming out of the programs. The Ministry has to look at the HR issues regarding PSWs and do something about it. That’s province-wide.”* [CR2]

*“*…*in a climate where there are PSW shortages, we have further difficulty in finding people that are bilingual who have a car, who can travel distances to work...”* [CR2]

*“But we’re at a point where we’ve got to start thinking outside the box. And we’re hopeful, we’ve been advocating that the province would look at maybe some type of apprenticeship model that, you know, we could actually pay them while they’re in school.”* [CR8]

iii. **Funding Limitations and Opportunities**

*“[Community support services are] treated as less essential to other healthcare offerings despite their value and ability to offer high level care at the same or lower cost than if they were to be placed in institutional long-term care.”* [CR1]

*“…if seniors have an accident, if they don’t take their proper medication or whatever, they end up in the hospital and then they wait for a LTCH bed… if we were to reduce the base CSS offerings…They will either go to the hospital or they’ll become high risk. Yet [CSS] is at the cheapest end. It takes very little investment, but may avoid a fall, or a bunch of things and then they don’t tax the rest of the health system.”* [CR12]

## Abbreviations

ADP: Adult Day Program
ALC: Alternate Level of Care
ALS: Assisted Living Services
CSS: Community Support Services
DSW: Developmental Services Worker
LTC: Long-Term Care
LTCH: Long-Term Care Home
MOHLTC: Ministry of Health and Long-Term Care
OECD: Organisation for Economic Co-operation and Development
NFP: Not-For Profit
PSW: Personal Support Worker
RGI: Rent Geared to Income
RN: Registered Nurse
SH: Supportive Housing
TCLHIN: Toronto Central Local Health Integration Network
WHO: World Health Organization

## Footnotes

1 Note – internal refers to residents of the campus and external means either extending services out into the community (e.g., Meals on Wheels), or bringing local community onto the campus for programs and services (e.g., Day Programs).

2 Life lease housing properties are not owned by residents but rather provide the right to occupy the property and an “interest” in that property in exchange for a lump sum payment up-front, with monthly maintenance fees and property tax payments. Residents own their own units but must sell back to the organization when moving or in the event of death [58].
